# Mediating effect of body fat percentage in the association between ambient particulate matter exposure and hypertension: a subset analysis of China hypertension survey

**DOI:** 10.1186/s12889-023-16815-0

**Published:** 2023-10-02

**Authors:** Yan Xue, Jin Li, Yu-Nan Xu, Jia-Sheng Cui, Yue Li, Yao-Qiong Lu, Xiao-Zhi Luo, De-Zhao Liu, Feng Huang, Zhi-Yu Zeng, Rong-Jie Huang

**Affiliations:** 1 Department of Cardiology, The First Affiliated Hospital of Guangxi Medical University, No.6 Shuangyong Road, Nanning, 530021 China; 2 Guangxi Key Laboratory of Precision Medicine in Cardio-Cerebrovascular Diseases Control and Prevention, Nanning, China; 3 Guangxi Clinical Research Center for Cardio-Cerebrovascular Diseases, Nanning, China; 4 Department of Medical Research, The First Affiliated Hospital of Guangxi Medical University, Nanning, China

**Keywords:** Particulate matter, Body Fat percentage, Mediating Effect, Hypertension, Population-attributable risk fraction

## Abstract

**Background:**

Hypertension caused by air pollution exposure is a growing concern in China. The association between air pollutant exposure and hypertension has been found to be potentiated by obesity, however, little is known about the processes mediating this association. This study investigated the association between fine particulate matter (aerodynamic equivalent diameter ≤ 2.5 microns, PM2.5) exposure and the prevalence of hypertension in a representative population in southern China and tested whether obesity mediated this association.

**Methods:**

A total of 14,308 adults from 48 communities/villages in southern China were selected from January 2015 to December 2015 using a stratified multistage random sampling method. Hourly PM2.5 measurements were collected from the China National Environmental Monitoring Centre. Restricted cubic splines were used to analyze the nonlinear dose-response relationship between PM2.5 exposure and hypertension risk. The mediating effect mechanism of obesity on PM2.5-associated hypertension was tested in a causal inference framework following the approach proposed by Imai and Keele.

**Results:**

A total of 20.7% (2966/14,308) of participants in the present study were diagnosed with hypertension. Nonlinear exposure-response analysis revealed that exposure to an annual mean PM2.5 concentration above 41.8 µg/m^3^ was associated with increased hypertension risk at an incremental gradient. 9.1% of the hypertension burden could be attributed to exposure to elevated annual average concentrations of PM2.5. It is noteworthy that an increased body fat percentage positively mediated 59.3% of the association between PM2.5 exposure and hypertension risk, whereas body mass index mediated 34.3% of this association.

**Conclusions:**

This study suggests that a significant portion of the estimated effect of exposure to PM2.5 on the risk of hypertension appears to be attributed to its effect on alterations in body composition and the development of obesity. These findings could inform intersectoral actions in future studies to protect populations with excessive fine particle exposure from developing hypertension.

**Supplementary Information:**

The online version contains supplementary material available at 10.1186/s12889-023-16815-0.

## Introduction

Hypertension is a globally prevalent condition and contributes to millions of deaths from cardiovascular and renal diseases worldwide [1]. Over the past 30 years, new cases of hypertension have mainly been distributed in East Asia, the Pacific, and sub-Saharan Africa, according to surveillance data from 200 countries worldwide [1]. With the number of hypertensive individuals doubling over the past 20 years, China has the highest absolute burden of hypertension in the world, with over 240 million people affected [2, 3]. Furthermore, China is one of the regions worldwide with a great increase in average systolic and diastolic blood pressure over the past 40 years [4]. The increasing burden of hypertension has led to excessive mortality in the Chinese population [5–7]. Therefore, identifying and addressing controllable risk factors for hypertension is a priority.

Air quality in China poses serious challenges. A national survey showed that exposure to atmospheric emissions, especially fine particulate matter (aerodynamic diameter ≤ 2.5 μm, PM2.5), was strongly related to an increased risk of hospitalization for 7 major disease categories during 2013–2017 [8]. In addition, abundant epidemiological evidence suggests that long- or short-term exposure to high concentrations of ambient PM2.5 has increased the risk of hypertension in the past decade [9–14]. Possible reasons include the following: (a) the impact of PM2.5 exposure on vascular dysfunction and remodeling [15–17]; (b) possible systemic inflammation, oxidative stress, and altered neuroendocrine factors caused by PM2.5 exposure [18, 19]; and (c) other unclear drivers.

Inconsistent results have been shown for the dose-effect relationship between PM2.5 exposure and the risk of hypertension, as observed and measured in different surveys. For instance, based on a survey involving 43,745 7–18-year-old individuals seeking treatment in China, Zhang and his colleagues found that each 10 µg/m^3^ increase in environmental PM2.5 levels was associated with a 1.46-mmHg increase in systolic blood pressure (SBP) or a 45% higher risk of hypertension [14]. Xie et al. reported that an increase of 10 µg/m^3^ of PM2.5 was associated with an odds ratio (OR) of 1.010 for hypertension and a 0.569-mmHg increase in SBP in reproductive-age adults [20]. This inconsistent result may be related to the demographic characteristics or geographic heterogeneity of the subjects. More critically, potential mediators or modifiers of PM2.5-associated hypertension are important but still not fully recognized regarding the occurrence of hypertension.

Overweight and obesity have been observed to enhance susceptibility to air pollutant-related cardiovascular disease [21, 22]. Previous studies have found that obesity may mediate the relationship between air pollution and hypertension [21]. Unfortunately, these studies mainly focused on the traditional parameters of obesity, such as body mass index (BMI) and waist circumference; however, BMI does not distinguish between lean body mass and fat body mass, while adipose tissue is an important source of vasoconstrictor activators induced by air pollutants [23], suggesting that BMI may be insufficient for assessing the effects of air pollution on hypertension risk [24]. In this study, we focused on whether body fat percentage (BF%), an indicator that provides information on cardiovascular risk in low BMI conditions [25, 26], mediates the association between air pollution exposure and hypertension risk. The selection of potential mediators was based on the following evidence: (a) a prospective cohort study showing that exposure to air pollutants strongly affects BF% [27]; and (b) epidemiological evidence indicating that BF% is an independent risk factor for the onset of hypertension in postmenopausal women [28] and elderly individuals [29].

The purpose of this study was to assess the exposure-response relationship, population-attributable risk fraction, and potential mediators of the relationship between ambient PM2.5 exposure and hypertension risk in multiethnic adults in South China. Our research provides clinically accessible and optimized indicators for large-scale community health surveillance aimed at assessing the impact of air pollution exposure on hypertension risk and provides new insights into the development of public health policies to ameliorate the burden of hypertension in regions with high air pollution exposure.

## Materials and methods

### Study population

This population-based cross-sectional survey was part of The China Hypertension Survey [2, 30]. In this study, a nationally representative sample of Chinese community residents aged ≥ 15 years was obtained using a multistage stratified random sampling method.

**Fig. 1:**
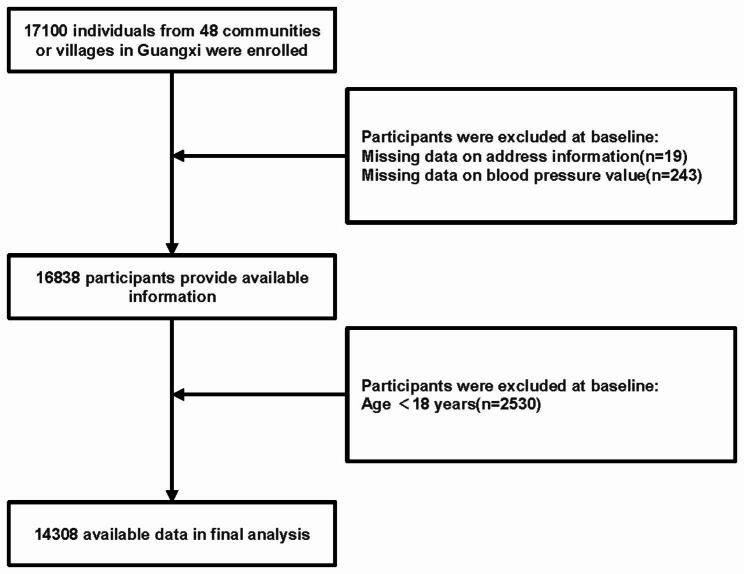
The flow chart of the study population selection

Given that environmental and biological differences among regions may corrode the association between air pollutant exposure and hypertension risk [3, 4], the inclusion of a homogeneous population from the same region as the study population was beneficial in minimizing this effect. We selected residents of the Guangxi Zhuang Autonomous Region as the major population to improve the robustness and scientific replicability of results for future investigations. A total of 17 100 residents (defined as individuals residing for 12 months or longer) from 48 communities or villages in this region were enrolled in this subset analysis. After exclusion (n = 262) due to missing information on blood pressure (BP) value or address, 16,838 individuals were eligible and completed the questionnaire. Participants aged ≥ 18 years (n = 14,308) were included in the final analysis. The participant recruitment and screening process is presented in Fig. 1. For each participant, the process of the survey was well-communicated and written informed consent was obtained. The Ethics Committee of The First Affiliated Hospital of Guangxi Medical University (Guangxi, China) approved the study (approval number: 2012[KY-E-017]). This study was conducted in strict accordance with the Declaration of Helsinki. This study has been registered on the Chinese Clinical Trials Registry (ChiCTR-ECS-14,004,641).

### Data collection

A standardized questionnaire was utilized to obtain information on demographic and socioeconomic characteristics. BMI was derived by dividing weight by the square of height (kg/m^2^). BF% was measured by a weight and body fat measurement device (V-Body HBF-371; OMRON Corporation, Tokyo, Japan), which was calibrated by the manufacturer. Participants underwent 3 times BP measurements using a professional BP monitor (HBP-1300, OMRON Corporation, Tokyo, Japan). Subsequently, the average value of the 3 BP measurements was utilized for analysis, as documented in a previous report [2].

### Outcome definitions

Hypertension was defined as meeting at least one of the following criteria: (a) an SBP ≥ 140 mmHg and/or a diastolic blood pressure (DBP) ≥ 90 mmHg; and (b) taking medication to lower BP within 2 weeks [31]. Overweight was defined as a BMI ≥ 24 kg/m^2^. The remaining variables not mentioned were defined in detail in a previous article [30].

### Assessment of PM2.5 exposure

In this study, 8 air quality monitoring stations located in Nanning (station code 1401-1408 A), 7 stations located in Liuzhou (station code 1870-1875 A), 4 stations located in Guilin (station code 1862-1865 A), 3 stations located in Yulin (station code 2509-2511 A), 3 stations located in Qinzhou (station code 2502-2504 A), 3 stations located in Hechi (station code 2516-2518 A) and 2 stations located in Laibin (station code 2519-2520 A) were selected for data collection, and hourly PM2.5 measurements from January 1, 2013, to December 31, 2015, were obtained from open source data on the Ministry of Ecology and Environment of the People’s Republic of China website (http://www.mee.gov.cn/).

The 24-hour average PM2.5 concentration was calculated based on hourly measurements, with at least 18 h of available measurements required for a natural day. The geographic locations of the 48 communities or villages were obtained by questionnaire. We matched places of residence with PM2.5 data from the nearest air quality monitoring station according to distance on the Auto Navi Map.

### Statistical analysis

SPSS 22.0 (IBM Corp., Armonk, N.Y., USA) and R software 4.1.3 (R Foundation for Statistical Computing, Vienna, Austria) were utilized to perform statistical analysis, and a two-tailed *p* value < 0.05 was considered statistically significant. Continuous variables are expressed as the mean (± standard deviation) or median (interquartile range). Categorical variables were presented as numbers and percentages. Student’s t tests or Wilcoxon rank tests for continuous variables, and chi-square tests for categorical variables were applied to perform intergroup comparisons. Contemporaneous national census data were employed to establish an age-standardized prevalence of hypertension.

To determine the potential nonlinear association between PM2.5 exposure and hypertension risk, we built restricted cubic splines with knots calculated on the basis of the minimum Akaike information criterion (AIC) value as the previous report [32]. Owing to the multistage random sampling resulting in the clustering of participants with a multilevel structure, we conducted a two-level logistic regression model to estimate the association between 12-month average PM2.5 exposure and the risk of hypertension in adults in southern China, where individuals were defined as first-level groups and communities and villages were defined as second-level groups, as described in previous research [33]. In addition, the following baseline variables were considered potential confounding variables for the occurrence of hypertension: demographic, socioeconomic, and lifestyle characteristics, such as sex, age, race, smoking status, family history of hypertension, BMI, alcohol consumption, education level, and residence type. PM2.5 exposure was categorized into quartiles (Q1: ≤ 25th percentile, Q2: [between the 25th and 50th percentile], Q3: [between the 50th and 75th percentile] and Q4: > 75th percentile), and the minimal PM2.5 exposure group (Q1) was used as a reference to assess the association between exposure and hypertension risk. In the sensitivity analysis, we explored the mean PM2.5 exposure levels at 1, 2, and 3 years and their associations with the risk of hypertension. We further matched identifiable variables (age, sex, and BMI) using the propensity score matching method.

### Estimation of the attributable hypertension burden

Population-attributed risk fraction (PAR%) and attributable cases were used to estimate the increased hypertension burden attributable to environmental PM2.5 exposure. The PAR% was calculated using the following equation: PAR% = P (OR-1) / [1 + P (OR-1)] *100%, where P is the hypertension prevalence among individuals exposed to high annual average PM2.5 concentrations. The standard error of the PAR% was calculated using the delta method. Cases of hypertension attributed to PM2.5 exposure were estimated as follows: Attributable cases = Overall cases * PAR%.

### Mediation analysis

Experimental and epidemiological findings indicate that obesity and metabolic pathways that are perturbed by air pollution exposure may lead to hypertension [34–38]; thus, they could be considered mediators in the causal pathways. Obesity indicators such as BMI have been used to explain the biological mechanisms linking air pollution exposure and dyslipidemia risk [39]; and glucose homeostasis [40]. The mediating effects of obesity in the relationship between air pollution exposure and hypertension risk, particularly the indicators with the strongest mediating effects, are still poorly understood. Causal mediation analysis is a typical technique used to evaluate mediating effects in recognizing causal effects [41, 42]. Compared to classical methods, the modified causal mediation analysis approach of Imai and Keele is considered more appropriate for use in conditions where the independent variable is nonlinearly associated with the dependent variable [43, 44].

In the present study, we treated obesity parameters, including BMI, BF%, waist circumference, and weight as potential mediators of the pathway from air pollutant exposure to hypertension. We followed a standard procedure for mediation analyses [45] involving three main steps to perform a series of linear and multivariable logistic regressions adjusted for sex, age (regarded as a categorical variable), ethnicity, current smoking status, BMI, alcohol consumption, education, urbanity, locations, and family history of hypertension. In the initial step, we conducted an evaluation to examine the associations between exposure (ambient PM2.5 concentration over a period of 1–3 years) and the outcome (hypertension). Subsequently, we assessed the association of exposure with mediators and mediators with outcomes separately. In the third step, the potential mediators and exposure were included in a linear regression to examine the direct or indirect effects of PM2.5 exposure on hypertension risk. Mediation models were fitted using a mediation package in the R software.

## Results

### Descriptive statistics

**Fig. 2:**
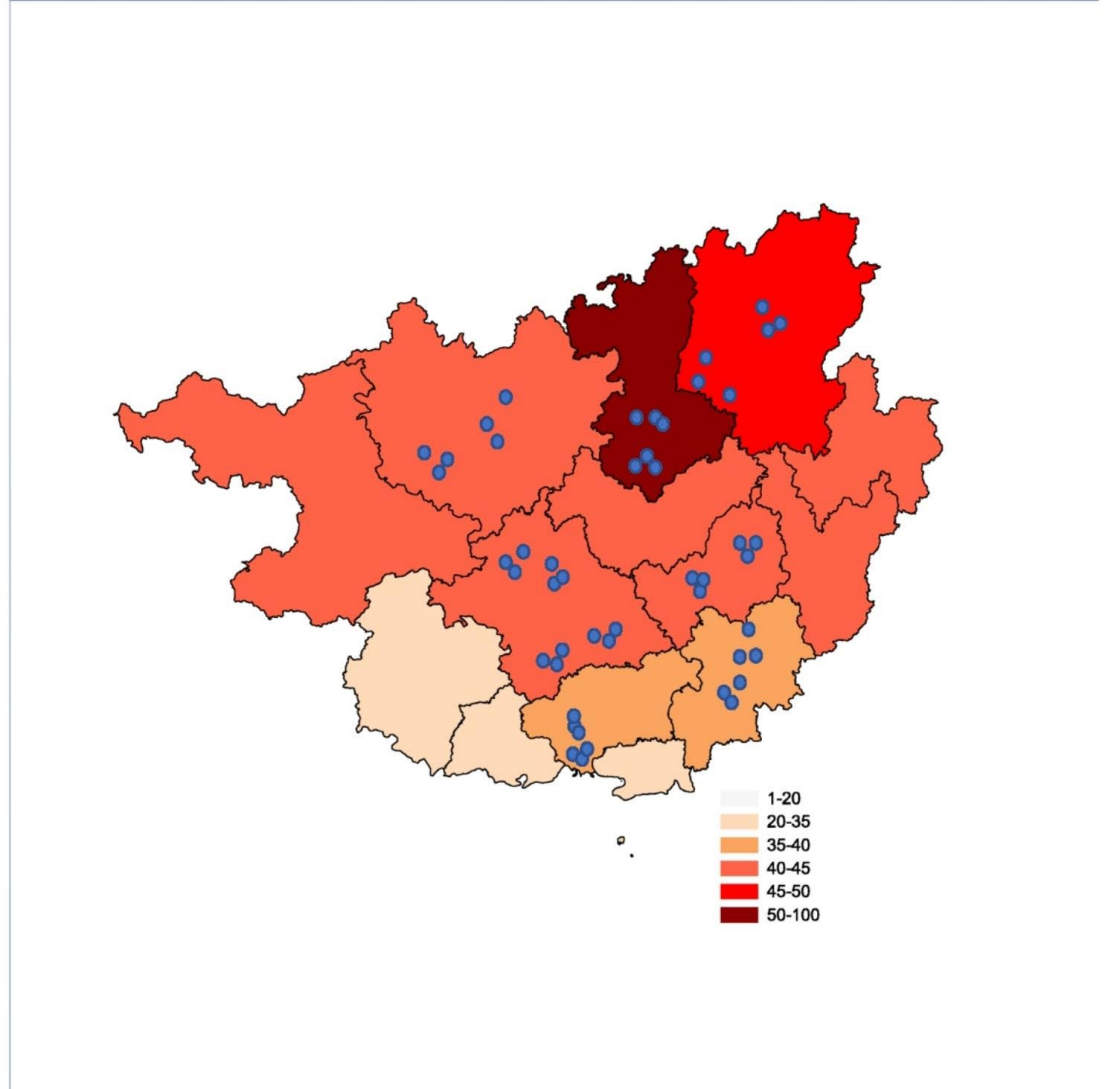
Geographic location and 12-month average PM2.5 exposure levels of 14 308 participants from 48 communities or villages in south China in 2015. The blue dot represented a single selected community or village

A total of 14,308 adults (7516 females and 6792 males, 57.6% from urban areas and 42.4% from rural areas) in South China were included in the final analysis. The detailed baseline characteristics of the participants are presented in Table 1. The spatial distribution of the participants and their average annual PM2.5 exposure levels are shown in Fig. 2.

**Table 1:** Characteristics of 14,308 Participants in this study

Characteristic	Overall (*n* = 14,308)	Control group (*n* = 11,342)	HT group (*n* = 2966)	P value
Age, y, n (%)				< 0.001
≤ 45	7181(50.2)	6871(60.6)	310(10.5)	
46–59	2978(20.8)	2281(20.1)	697(23.5)	
≥ 60	4149(29.0)	2190(19.3)	1959(66.0)	
12-mo Average PM2.5 µg/m^3^	42.0 ± 4.6	41.2 ± 4.5	44.9 ± 5.0	< 0.001
Sex, n (%)				0.651
Female	7516(52.5)	5947(52.4)	1569(52.9)	
Male	6792(47.5)	5395(47.6)	1397(47.1)	
Body mass index, Kg/m^2^	22.3 ± 3.2	22.0 ± 3.0	23.3 ± 3.5	< 0.001
Body fat percentage, %	25.07 ± 7.97	23.91 ± 7.65	29.45 ± 7.62	< 0.001
Waist circumference, cm	78.76 ± 9.24	77.70 ± 8.84	82.83 ± 9.60	< 0.001
Systolic blood pressure, mm Hg	126.9 ± 19.0	119.3 ± 10.3	155.9 ± 16.2	< 0.001
Diastolic blood pressure, mm Hg	74.4 ± 9.7	72.0 ± 7.6	83.5 ± 11.3	< 0.001
Heart rate, bpm	77.2 ± 9.7	76.7 ± 9.1	79.0 ± 11.4	< 0.001
Education level, n (%)				< 0.001
Primary school or below	6103(42.7)	3999(35.3)	2104(70.9)	
Junior high school	6049(42.3)	5358(47.2)	691(23.3)	
High school	1689(11.8)	1550(13.7)	139(4.7)	
College or above	467(3.3)	435(3.8)	32(1.1)	
Ethnicity, n (%)				< 0.001
Han nationality	6730(47.0)	5464(48.2)	1266(42.7)	
Other nationality	7578(53.0)	5878(51.8)	1700(57.3)	
Household registration, n (%)				0.005
Urban	8235(57.6)	6461(57.0)	1774(59.8)	
Rural	6073(42.4)	4881(43.0)	1192(40.2)	
City, n (%)				< 0.001
Nanning	3896(27.2)	3013(26.6)	883(29.8)	
Guilin	697(4.9)	450(4.0)	247(8.3)	
Liuzhou	1897(13.3)	1473(13.0)	424(14.3)	
Yulin	2025(14.2)	1677(14.8)	348(11.7)	
Qinzhou	1996(14.0)	1692(14.9)	304(10.2)	
Hechi	1789(12.5)	1391(12.3)	398(13.4)	
Laibin	2008(14.0)	1646(14.5)	362(12.2)	
Ever smoked, yes, n (%)	2147(15.0)	1600(14.1)	547(18.4)	< 0.001
Drinking, yes, n (%)	2300(16.1)	1706(15.0)	594(20.0)	< 0.001
Family history of hypertension, yes, n (%)	1210(8.5%)	948(8.4)	262(8.8)	0.408

In the present study, the participants had a mean SBP of 126.9 mmHg and a mean DBP of 74.4 mmHg, with a crude hypertension prevalence of 20.7% (Table 1). Patients with hypertension were more likely to be older than 60 years, have been exposed to higher 12-month average ambient PM2.5 levels, and had higher BMI, BF%, waist circumference, and weight compared to controls.

### The nonlinear relationship between ambient PM2.5 exposure and hypertension risk

**Fig. 3:**
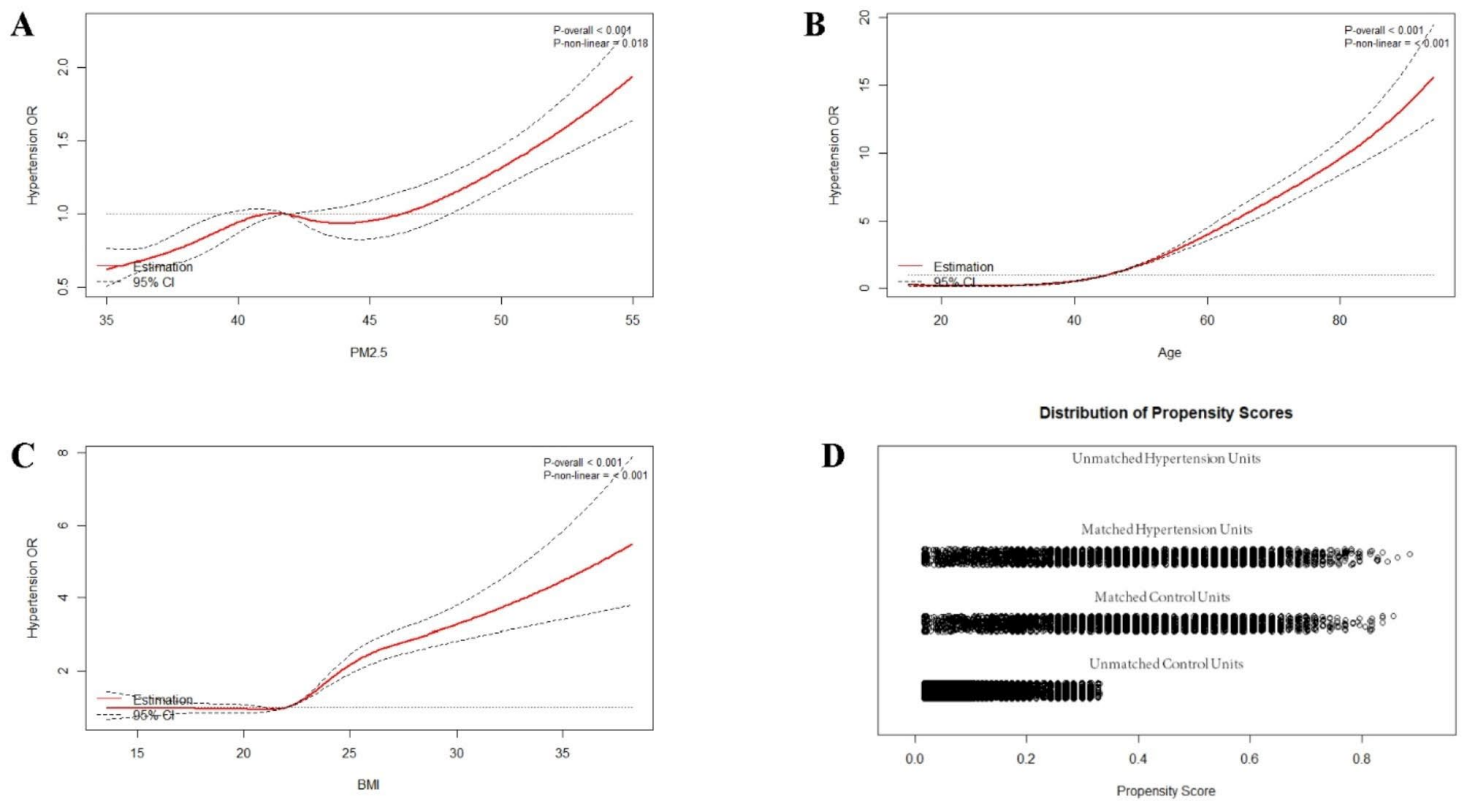
Concentration-response curves for the effects of ambient PM2.5(**A**), age (**B**), and body mass index (**C**) on hypertension. OR means odds ratio. Distribution of Propensity score (**D**) of participants before and after matching

As shown in Fig. 3, we used restricted cubic splines to flexibly model and visualize the relationship between annual PM2.5 exposure, age, and BMI and the risk of hypertension in the southern Chinese population. As presented in Fig. 3A, we observed a nonlinear relationship between the average annual PM2.5 concentration and the risk of hypertension (nonlinear *p* = 0.018). The relationship between PM2.5 exposure and the risk of hypertension exhibited an amplified slope when the concentration exceeded 41.8 µg/m^3^ (Fig. 3A). In addition, when considered as a continuous variable, age and BMI were significantly and nonlinearly associated with the risk of hypertension (nonlinear *p* < 0.001, Fig. 3B C).

### Impact of annual PM2.5 exposure on the risk of hypertension in South China

For the assessment of the effect size of ambient PM2.5 exposure affecting the risk of hypertension, logistic regression analyses were performed with a minimum 25% PM2.5 exposure or an annual average below 41.8 µg/m^3^ as references, adjusting for potential confounders including sex, age (considered as a categorical variable), race, current smoking status, BMI, alcohol consumption, educational level, urbanization, location, and family history of hypertension. The results showed that each 5 µg/m^3^ increase in annual mean PM2.5 exposure was associated with a 4% higher risk of hypertension (OR = 1.040, 95% confidence interval (CI) = 1.007–1.062). Furthermore, individuals exposed to annual average PM2.5 concentrations above 41.8 µg/m^3^ was associated with an 18.3% increased risk of hypertension compared to those with lower average exposure (OR = 1.183, 95%CI = 1.165–1.212). As mentioned previously, exposure to higher mean annual PM2.5 concentration was associated with an increased risk of individual hypertension in a nonlinear pattern with ORs of 1.038 (95%CI = 1.006–1.058) (Q2 vs. Q1), 1.084 (95%CI = 1.071–1.102) (Q3 vs. Q1), and 1.421 (95%CI = 1.308–1.561) (Q4 vs. Q1), respectively (Table 2). Sensitivity analyses demonstrated that the estimates of hypertension risk associated with exposure to high ambient PM2.5 concentrations remained robust after propensity score matching for age, sex, and BMI (see Table S1 and Fig. 3D), consistent with previous reports [46]. Alternative analyses using 1-, 2-, and 3-year mean air pollution exposures consistently generated similar estimates (Table S2).

**Table 2:** Estimated Effects of Hypertension with Ambient PM2.5*

PM2.5, µg/m^3^	Effect Size (95% CI)	P value
Continuous variable		
Per 5-µg/m^3^ increase	1.040 (1.007–1.062)	< 0.001
Categorized by slope steeper point		
< 41.8 µg/m^3^	1.0 (Reference)	
≥ 41.8 µg/m^3^	1.183(1.165–1.212)	< 0.001
Categorized by quintiles		
Q1(< 37.92)	1.0 (Reference)	/
Q2(37.92–43.64)	1.038(1.006–1.058)	< 0.001
Q3(43.64–48.69)	1.084(1.071–1.102)	< 0.001
Q4(≥ 48.69)	1.421(1.308–1.561)	< 0.001

* Data were adjusted for sex, age (regarded as a categorical variable), ethnicity, current smoking status, BMI, alcohol consumption, education, urbanity, locations, and family history of hypertension

As presented in Table 3, in 9.1% (95% CI = 8.3-10.4%) or 270 patients (95% CI = 245–308) out of a total of 2966 patients, hypertension could be attributed to exposure at annual average PM2.5 concentrations higher than 41.8 µg/m^3^. Similar effects on the burden of hypertensive disease attributable to PM2.5 exposure were observed in the sex and age subgroups. Only 6% of participants with a BMI < 24 kg/m^2^ had hypertension that could be attributed to PM2.5 exposure, while this percentage was as high as 14.4% in those with a BMI ≥ 24 kg/m^2^.

**Table 3:** Estimated Hypertension Burden Attributable to ambient PM2.5(Categorized by a concentration of 41.8 µg/m^3^) *

Variable	No. of Subjects with Hypertension	Population-Attributable Fraction, % (95% CI)	Attributed Hypertension Cases, n (95% CI)
Overall	2966	9.1 (8.3–10.4)	270 (245–308)
Sex			
Female	1569	7.3(6.8–8.5)	115(107–133)
Male	1397	10.0(9.2–11.2)	140(129–157)
Age			
≤ 45	310	10.4(9.9–11.3)	32(31–35)
> 45	2656	7.6(7.1–8.2)	203(189–217)
BMI			
< 24	1794	6.0(5.7–6.3)	107(102–113)
≥ 24	1172	14.4(12.8–15.8)	169(150–186)

* Data were adjusted for sex, age (regarded as a categorical variable), ethnicity, current smoking status, BMI, alcohol consumption, education, urbanity, locations, and family history of hypertension

### Association between body composition, obesity, and PM2.5 exposure

Partial correlation coefficients were estimated to examine the correction among obesity parameters with annual mean PM2.5 concentrations and high blood pressure, as shown in Table S3. Furthermore, we evaluated the eligibility of 4 obesity indicators that should serve as mediators of the association between PM2.5 exposure and hypertension risk by multivariate linear and logistic regression. As shown in Table 4, increased PM2.5 levels per standard deviation (SD) were associated with higher SBP (β = 0.198, 95% CI = 0.102–0.294) and DBP (β = 0.250, 95% CI = 0.202–0.299). Higher PM2.5 was significantly associated with increased BMI (β = 0.173, 95% CI = 0.075–0.271), BF% (β = 0.242, 95% CI = 0.189–0.294), WC (β = 0.155, 95% CI = 0.134–0.177), and weight (β = 0.146, 95% CI = 0.125–0.167) in residents.

**Table 4:** Associations between exposures (PM2.5), mediators (BMI, BF%, WC and weight), and the outcome (hypertension)

	β (95% CI)	P value
Exposure^**a**^ (PM2.5) to outcome^b^		
SBP	0.198(0.102–0.294)	< 0.001
DBP	0.250(0.202–0.299)	< 0.001
Exposure^**a**^ (PM2.5) to mediators^b^		
BMI	0.173(0.075–0.271)	< 0.001
BF%	0.242(0.189–0.294)	< 0.001
WC	0.155(0.134–0.177)	< 0.001
Weight	0.146(0.125–0.167)	< 0.001
Mediators to outcome (SBP)^**b**^		
BMI	0.528(0.514–0.542)	< 0.001
BF%	0.547(0.539–0.554)	< 0.001
WC	0.182(0.178–0.185)	< 0.001
Weight	0.146(0.142–0.149)	< 0.001
Mediators to outcome (DBP) ^**b**^		
BMI	0.219(0.187–0.251)	< 0.001
BF%	0.955(0.950–0.960)	< 0.001
WC	0.099(0.092–0.106)	< 0.001
Weight	0.085(0.078–0.092)	< 0.001
	Odds ratio (95% CI)	P value
Mediators to outcome (hypertension)c		
BMI	1.130(1.115–1.144)	< 0.001
BF%	1.145(1.138–1.151)	< 0.001
WC	1.040(1.035–1.056)	< 0.001
Weight	1.028(1.011–1.031)	< 0.001

*PM2.5: particulate matter with aerodynamic diameter < 2.5 μm; BMI: body mass index; BF% : body fat percentage; WC: waist circumference; SBP: systolic blood pressure; DBP: diastolic blood pressure

^a^ Associations reflect change in outcome measure (association estimate (β)) scaled to 1 standard deviation of prior 1-year average ambient PM2.5 with 4.0 µg/m^3^

^b^ Linear regression models were adjusted for sex, age (regarded as a categorical variable), ethnicity, current smoking status, BMI, alcohol consumption, education, urbanity, locations, and family history of hypertension

^c^ Multivariate logistic regression model was adjusted for sex, age (regarded as a categorical variable), ethnicity, current smoking status, BMI, alcohol consumption, education, urbanity, locations, and family history of hypertension

### Association between body composition, obesity, and hypertension

In the models assessing the association between mediators and hypertension risk, we observed that exposure to each 1 SD of higher BMI (OR = 1.130, 95% CI = 0.075–0.271), BF% (OR = 1.145, 95% CI = 1.138–1.151), WC (OR = 1.102, 95% CI = 1.092–1.113), and weight (OR = 1.086, 95% CI = 1.076–1.096) was significantly associated with an increased hypertension risk, after adjusting for covariates mentioned in the previous section (Table 4). Moreover, consistent associations were observed between the mediators and both SBP and DBP measurements.

Furthermore, all 4 obesity parameters were associated with exposure to high annual mean PM2.5 concentrations and an increased risk of hypertension and corroded the association between PM2.5 exposure and the risk of hypertension when included in the model, as shown in Table 5.

**Table 5:** Association between high PM2.5 exposure and hypertension adjusted for various obesity parameters

Model	OR	95% Confidence Interval	p value
Model 1			
Unadjusted	1.48	1.44–1.51	< 0.001
Model 2*			
Adjusted	1.33	1.30–1.37	< 0.001
Model 3			
Model 2 + BMI	1.18	1.17–1.21	< 0.001
Model 4			
Model 2 + BF%	1.11	1.09–1.12	< 0.001
Model 5			
Model 2 + WC	1.28	1.25–1.31	< 0.001
Model 6			
Model 2 + Weight	1.32	1.28–1.35	< 0.001

* Model 2: Multivariable logistic regression adjusted for age adjusted for sex, age (regarded as a categorical variable), ethnicity, current smoking status, alcohol consumption, education, urbanity, locations, and family history of hypertension. BMI: body mass index; BF%: body fat percentage; WC: waist circumference

### Mediation analysis

**Fig. 4:**
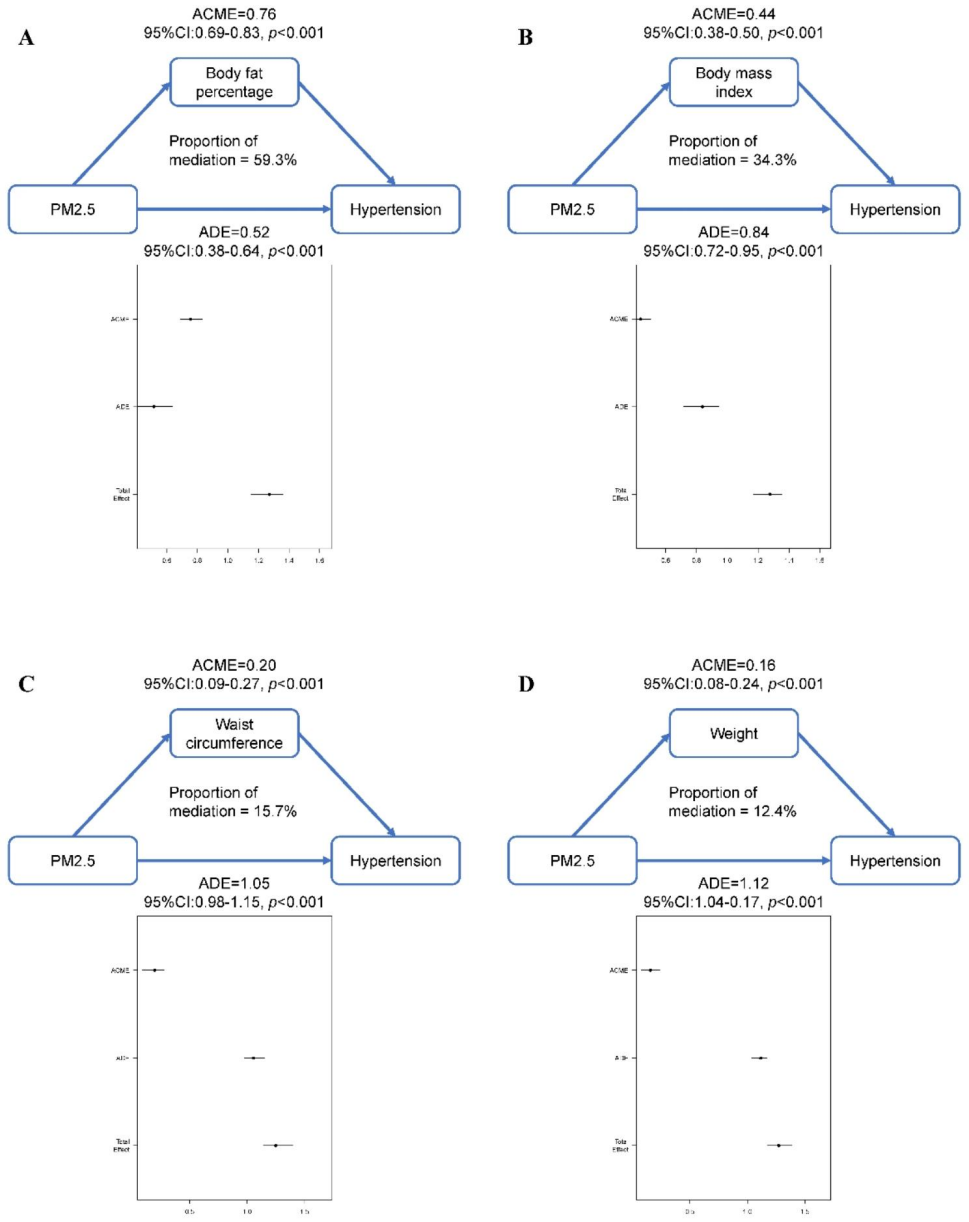
It was estimated that body fat percentage (**A**) mediated the highest association between PM2.5 concentration and hypertension, over body mass index (**B**), waist circumference (**C**), and weight (**D**). ACME, average causal mediation effects. ADE, average direct effects

We further conducted a mediation analysis to explore the potential mediating effects of obesity and body composition indicators on the association between PM2.5 exposure and hypertension risk. Mediation analyses adjusting for potential confounders showed that 59.3% (95% CI = 52.4-68.0%, *p* < 0.001) of the excess risk of hypertension associated with exposure to high annual average PM2.5 concentrations was attributed to elevated BF% (Fig. 4A), while 34.3% (95% CI = 29.5-41.3%, *p* < 0.001) of the excess risk was mediated by elevated BMI, 15.7% (95% CI = 8.1-20.0%, *p* < 0.001) was mediated by elevated waist circumference and 12.4% (95% CI = 6.7-17.2%, *p* < 0.001) was mediated by elevated weight (Fig. 4B and D).

## Discussion

The study findings revealed a non-linear association between high ambient concentrations of PM2.5 and an increased risk of hypertension. Additionally, it was estimated that 9.1% of the burden of hypertension could be attributed to exposure to an annual average PM2.5 concentration exceeding 41.8 µg/m^3^. Importantly, this effect appeared to be largely mediated by an increase in body fat percentage (BF%).

The results of this study were consistent with those of previous studies. A prospective cohort study that included 74,880 registered nurses in 11 states in the United States demonstrated a 4% higher risk of hypertension for every 5 µg/m^3^ increase in the 24-month average PM2.5 concentration [22]. In addition, an increase of 10 µg/m^3^ in the PM2.5 concentration was observed to be associated with an 11% increased risk of hypertension in a large-scale cross-sectional study in China [47]. A similar result was found in another cohort study including 59,456 adults in China (HR = 1.11; 95% CI = 1.05–1.17) [48].

However, other studies reported different findings. A study that included 7 European air pollution effect cohorts totaling 41,072 participants who did not report hypertension at baseline indicated that each 5 µg/m^3^ increase in the PM2.5 exposure level resulted in a 22% increased risk of hypertension [11]. Moreover, Trenton Honda et al. reported a 13% increased risk of hypertension for every interquartile range (equal to 3.98 µg/m^3^) increase in the ambient PM2.5 concentration [49]. These controversial results, although explainable or partially explainable by differences in atmospheric pollutant composition [50] and population demographic characteristics [51], indicate that the hypothesis that the exposure-response relationship between PM2.5 exposure and hypertension risk may be affected by mediators remains reasonable, and the direct and mediating effects of PM2.5 exposure and hypertension risk warrant further exploration.

Moreover, we conducted an assessment of the hypertension burden that could be attributed to PM2.5 exposure in the overall population and specific populations. Compared with many previous studies that used ORs or relative risks to demonstrate the relationship between PM2.5 exposure and hypertension risk, we combined the OR of PM2.5 exposure and hypertension risk with the percentage of the population exposed to high PM2.5 concentrations to provide a more comprehensive and understandable perspective. It was estimated that 9.1% of the hypertension burden could be attributed to exposure at an annual average PM2.5 concentration over 41.8 µg/m^3^. Similar results were observed in the subgroup analysis stratified by sex and age. However, we found that 14.4% of the hypertension burden could be attributed to exposure to high PM2.5 concentration in individuals with a BMI ≥ 24 kg/m^2^, whereas only 6.0% of the hypertension burden could be attributable to exposure to high PM2.5 concentration in those with a BMI < 24 kg/m^2^. BMI, a traditional indicator of obesity, is thought to be associated with PM2.5 exposure [35, 52]. More importantly, obesity is likewise considered an independent risk factor for hypertension; thus, we hypothesized that obesity may mediate the association between PM2.5 exposure and hypertension risk. To assess the potential mediating effect in a multidimensional manner, we used different obesity parameters, including BF%, BMI, waist circumference, and weight. The results of the mediation analysis demonstrated that BF%, rather than BMI, appeared the strongest mediating effect, explaining approximately 60% of the total effect of PM2.5 exposure on hypertension risk.

BMI has been criticized as an indicator of obesity for not reflecting body composition characteristics and therefore providing insufficient warning of disease risk. BF% represents a sensitive indicator of metabolic characteristics and is associated with lower insulin sensitivity, a larger subcutaneous adipocyte size, and central fat distribution in obese patients, with this effect considered independent of BMI [53, 54]. A clinical trial aiming to evaluate the effects of dietary interventions on the mobilization of fat storage pools found that a Mediterranean low-carbohydrate dietary pattern reduced the BF% and the total amount of visceral adiposity, with this change in lipid traits being independent of a given BMI [55]. An elevated BF% was observed in individuals with exposure to ambient fine particulate matter [56, 57]. A cross-sectional study of people aged ≥ 65 years in Taiwan showed that increased PM2.5 exposure was strongly associated with higher BF% and lower skeletal muscle mass [58]. Cai and his colleagues examined the significant positive associations between ambient air pollutant exposure and elevated BF% in participants from the UK Biobank, with the strongest associations between PM2.5 concentrations and the amount of fat at the trunk and viscera [59]. Evidence from experimental and interventional studies suggests that BF%, indicative of adipose tissue distribution, appears to possess the potential to better reflect PM2.5 exposure-associated hypertension risk than BMI. However, its mediating effect on PM2.5 exposure-associated hypertension and its potential biological mechanisms remain unknown. Limited evidence suggests that a high BF% may be a consequence of environmental and genetic susceptibility and their interaction-driven systemic inflammatory state, adipokine imbalance, and proinflammatory transformation of the gut microbial profile [60, 61], which appears to explain its mediating effect on air pollutant-induced blood pressure elevation since recent metabolomic, epigenetic, and toxicological studies from obese humans or other mammals have supported air pollutants as a critical environmental trigger for the mechanisms mentioned above [62–65]. Moreover, PM2.5 exposure experimentally attenuated compensatory antioxidant reserves [66], and enhanced intense vasoactive lipid release [67], which may partially explain the direct causation of hypertension. Additionally, exposure to fine particulate matter enhances physiological contractile mechanisms mediated by perivascular adipose tissue and corrodes the sensitivity of the vessel to diastolic agents. Zhou et al. reported that mice exposed to high concentrations of PM2.5 had upregulated expression of adipokines and oxidative stress-related molecules in aortic perivascular adipose tissue. Isolated aortic ring diastolic experiments showed that exposure to air pollutants inhibited the response of the aorta to relaxation agonists (acetylcholine and sodium nitroprusside). Overexpression of extracellular superoxide dismutase reversed this effect, highlighting the possibility of targeting excessive adipose tissue-mediated oxidative stress to prevent air pollutant exposure-induced hypertension [68].

The mediation analysis we performed provide new insights for understanding the mechanisms linking air pollutant exposure and hypertension risk and for proposing new health policies. First, our findings provide early warning indicators available to community-based large-scale health surveillance programs for monitoring intermediate processes between air pollution exposure and hypertension risk. Second, our findings support the rationale of BF% assessments for seeking population-level solutions for hypertension. Finally, these results support the hypothesis that targeting the improvement of unhealthy body compositions will reduce the average blood pressure of residents living in regions with severe air pollution. Further studies are needed to identify the potential effects of mutual interactions among ambient fine particulate matter exposure, body composition, and blood pressure.

Some limitations in this study should be acknowledged. Firstly, as a cross-sectional study, it is unable to establish a causal association between PM2.5 exposure and the risk of hypertension, as well as the mediating role of body composition and obesity in this association. Further study with a long-term follow-up is urgently warranted to confirm our findings. Second, we did not assess longer-term PM2.5 exposure beyond 3 years because no national air pollution detection system was established in China before 2013; however, previous studies have found that 1-year average PM2.5 exposure levels have reliable predictive efficacy for hypertension risk [69, 70]. Third, we failed to assess individual PM2.5 exposure, which could lead to an inability to measure individual health hazards; however, we collected measurements from all monitors at regional air quality monitoring stations and selected values from the nearest monitor, and this approach could reflect and compliment the spatial variability of individual-level exposure. Finally, our results were analyzed from a population in southern China, which may limit their applicability in other regions.

In conclusion, we find a nonlinear relationship between mean annual PM2.5 exposure levels and the risk of hypertension in a representative population sampled by multistage stratified randomization. Moreover, 9.1% of the burden of hypertension could be attributed to exposure to annual mean PM2.5 concentrations above 41.8 µg/m^3^ in the population dimension. Importantly, the observed association between PM2.5 exposure and hypertension appears to be predominantly mediated by alterations in body composition and obesity, adding information to support public health policy development and personal protection planning to reduce potentially harmful exposures.

### Electronic supplementary material

Below is the link to the electronic supplementary material.

Supplementary Material 1

Supplementary Material 2

Supplementary Material 3

## Data Availability

The full datasets used in this analysis are available from the corresponding author upon reasonable request.

## Non-standard Abbreviations and Acronyms

PM2.5: Particulate matter with aerodynamic equivalent diameter ≤ 2.5 μm
BP: Blood pressure
SBP: Systolic blood pressure
DBP: Diastolic blood pressure
PAR%: Population attributable risk fraction
PSUs: Primary sampling units
PPS: Probability Proportional to Size
BMI: Body mass index
BF%: Body fat percentage
